# “Why would we?” A qualitative study on COVID-19 vaccination decision making among Ukrainian economic female migrants in Poland

**DOI:** 10.3389/fpubh.2024.1380627

**Published:** 2024-08-13

**Authors:** Maria Ganczak, Pawel Kalinowski, Piotr Twardowski, Dominika A. Osicka, Oskar Pasek, Łukasz Duda-Duma, Johanna P. M. Vervoort, Michael Edelstein, Marta Kowalska

**Affiliations:** ^1^Department of Infectious Diseases, Collegium Medicum, University of Zielona Góra, Zielona Góra, Poland; ^2^Department of Hygiene and Epidemiology, Medical University of Lublin, Lublin, Poland; ^3^Department of Epidemiology, University Medical Center Groningen, University of Groningen, Groningen, Netherlands; ^4^Student Research Group, Collegium Medicum, University of Zielona Góra, Zielona Góra, Poland; ^5^Department of Health Sciences, University Medical Center Groningen, University of Groningen, Groningen, Netherlands; ^6^Azrieli Faculty of Medicine, Bar-Ilan University, Safed, Israel

**Keywords:** vaccine, Ukrainian migrants, COVID-19, females, Poland

## Abstract

**Background:**

Ukraine has one of the lowest COVID-19 vaccination rates in Europe. This may pose a significant epidemiological risk in the context of the refugee crisis and the fact that, since 2020, SARS-CoV-2 has been spreading and changing globally.

**Objective:**

To evaluate determinants of vaccination decision making among Ukrainian female migrants (UFMs).

**Methods:**

A qualitative study with 45 UFMs was conducted between December 2021 and January 2022. UFMs, from 2 Polish provinces, differing in age, education and length of stay were invited with the use of the snowball technique. Using a semi-structured topic guide, eight focus groups were conducted in person, recorded and transcribed. Thematic, qualitative analysis was made; key themes which emerged from the data (with the help of the Working Group Determinants of Vaccine Hesitancy Matrix), were related to contextual, individual/group and contextual vaccine/vaccination-specific influences.

**Results:**

Mothers were found to be playing a crucial role in children and adolescent COVID-19 vaccine decision-making process. Universal trust in the Polish healthcare system and vaccination procedures, employer requirements and willingness to preserve jobs, desire to get back to normal and social influences were paramount prerequisites to let UFMs make a decision to get vaccinated. However, COVID-19 vaccines also faced backlash among UFMs. Negative experiences with vaccines provided in Ukraine, doubts about the rapid vaccine development, combined with lack of confidence in vaccine safety, specifically regarding child vaccination, might have a bearing on UFMs’ decision about declining COVID-19 vaccine while on migration. Discrimination through HCWs during vaccination visits was also reported. Corrupted Ukrainian healthcare system, which facilitates proof forgery regarding vaccination certificates, could act as a negative influencer of UFMs’ vaccine decision-making.

**Conclusion:**

The results provide the novel information, expressed in economic UFMs’ own words. Findings show that influencers of the decision-making process regarding the COVID-19 vaccination are complex and polarized; elements of hesitancy may persist after migration. Any continuation of UFMs’ vaccination with COVID-19 vaccine should be subject to designing accessible information to address modifiable demotivators of the vaccine decision-making process identified in this study.

## 1 Introduction

As of 10 January 2024, the COVID-19 pandemic has caused 773,448,535 cases and 6,991,829 confirmed deaths, making it one of the deadliest epidemics documented (1). While the acute phase of the COVID-19 pandemic has subsided, the virus continues to spread and to mutate into new variants (2). Safe and effective COVID-19 vaccines were widely deployed from December 2020. In 2021 alone, they saved an estimated 14.4 million lives worldwide (3, 4).

In Ukraine the COVID-19 immunization program started on 24 February 2021. One year after vaccines became available, the COVID-19 vaccination rate in Ukraine was 38% for the primary course and only 1.7% of the eligible population received a booster dose (5). There are several reasons behind low vaccination rates among Ukrainians, including healthcare-related factors such as delayed introduction of COVID-19 vaccines and shortages of supply (6, 7) and pre-existing dissatisfaction with the health-care system and services, and recognized corruption within the healthcare system fueling distrust for authorities and institutions (8, 9). Other factors, such as concerns about the safety of vaccines due to the fast development and deployment, as well as widespread misinformation regarding COVID-19, have also likely come into play (6, 10–12). Anti-vaccination movements have become increasingly active in Ukraine since the beginning of the 21st century (13, 14), and vaccine hesitancy and refusal by health care workers (HCWs) is common (8, 9, 13).

Immigration from Ukraine to Poland is a well-established migration route with economic, political and social consequences. As a result of the Russian aggression against Ukraine in 2014, unfavorable economic conditions and high un-employment in Ukraine, economy-driven migration flow from Ukraine into Poland, both permanent and seasonal, started to increase in the 2010s. In brief, an economic migrant is a person who leaves their home country to live in another country with better working or living conditions (15). In 2018 alone, Poland issued 1.6 million immigration documents to Ukrainians (16). Since the onset of the war between Russia and Ukraine, Ukrainian refugees have been fleeing from areas involved in the fighting or those at risk of conflict (17). Notably, refugees are in the EU countries under temporary protection. According to Ukrainian data (5), two thirds of the Ukrainian migrants and refugees were estimated to have not received any COVID-19 vaccination. In the context of ongoing SARS-CoV-2 transmission, vaccinating this vulnerable migrant population should be a high priority for Poland. Vaccinating this population has proved challenging, with low levels of uptake. Previous surveys highlighted hesitancy toward vaccines in general and COVID-19 vaccines specifically among Ukrainians compared with other countries (18, 19). To the best of the authors’ knowledge, no scientific study has been published to date regarding the factors that may influence the decision to be vaccinated against COVID-19 among economic Ukrainian female migrants (UFMs) living in Poland. We aimed to examine these factors in order to inform tailored intervention to improve vaccine uptake in this group.

## 2 Materials and methods

### 2.1 Study population and sampling

The study population was recruited as the Polish component of RIVER-EU (*Reducing Inequalities in Vaccine uptake in the European Region – Engaging Underserved communities*), a broader study aimed at improving child and adolescent MMR/HPV vaccination among underserved communities in Europe (20). The detailed project methodology is described elsewhere (21). In brief, this study was carried out between December 2021 and January 2022. Recruitment took place in the capital cities of two Polish regions: Lublin (Lubelskie province), located in the south-east of Poland and neighboring Ukraine; and Zielona Góra (Lubuskie province), in the western part of Poland, neighboring Germany. Ukrainian-born individuals aged 15 and over, residing in Poland for a minimum of 6 months and a maximum of 10 years (i.e., a recent migrant), were eligible. We focused the study on females, because they tend to be the decision-makers for the family with regards to vaccines, according to the literature (22). At the time the study was conducted, COVID-19 vaccines were available free of charge for those aged 12 and over. Eligible individuals were invited to participate into RIVER-EU study through social media (Facebook), through adverts at job agencies and institutions employing UFMs, and through an organization recruiting students willing to start education at Polish high schools.

Prior to consenting, adult participants received information sheets in Polish and Ukrainian that explained the study objectives, the voluntary nature of the study and the right to withdraw from the research at any time. For teenagers, a consent form together with project information was sent to their legal guardian; an invitation for the teenager was included. On the interview day, informed consent was confirmed, both from the teenager and their legal guardian in accordance with the Polish Act of the Medical Profession (UZL). For each participant, prior to interview, we collected socio-demographics details (age, sex, education, residency in Ukraine/Poland, number of children), length of stay in Poland, and number of trips between Ukraine and Poland per year. Each participant received a PLN 100,00 (about 22 EUR) compensation.

### 2.2 Data collection

Eight 60–90 min face-to-face focus group discussions were conducted with UFMs. Discussions took place at local universities, and were facilitated by Polish/Ukrainian interpreters. UFMs were asked about their attitudes toward the COVID-19 vaccine, factors influencing immunization decision making, and their previous vaccination experiences. Interviews were pseudonymized, audio recorded and transcribed verbatim by the researchers; a professional interpreter checked the content for clarification and amendment.

### 2.3 Data analysis

Thematic analysis was used. Interviews were analyzed thematically by the researchers using the steps outlined by Braun and Clarke (23): data familiarization, coding and theme identification, refinement. One researcher team member conducted a similar study among Ukrainian migrants in Poland (10) and brought her experience to this study.

The researchers managed the data using software program ATLAS.ti version 24; analyses were conducted separately for adults and teenagers. Two teams of authors reviewed the transcripts independently analyzing their content and assigning initial codes to text fragments. The constructs from the WHO’s Determinants of Vaccine Hesitancy (24) were used to classify the prominent codes and elaborate on general themes related to factors influencing the COVID-19 vaccine decision-making process shared across all FGs transcripts. Intensive literature searches were performed, with the use of articles on qualitative research methods, textbooks chapters (25–27), and papers describing qualitative studies on vaccination beliefs and attitudes (28–33). Ongoing discussions via video-conferences and in person helped team members to critically review, discuss and refine initial coding and achieve a consensus regarding the final coding scheme. Codes were then arranged in sub-themes and themes. Deductive and inductive approaches were used to analyze which themes emerged from the qualitative data. In the end, illustrative excerpts from coded data were derived and classified within the appropriate thematic domain. Relevant quotes were collected and combined. Finally, by consensus among all research team members those UFMs’ statements which reflected themes the most were decided on to ensure adequate saturation, coherence, and reliability.

All researchers agreed on following key analysis themes presented in Supplementary Table S1.

### 2.4 Ethics considerations

The study was approved by the Institutional Ethics Committee of the University of Zielona Góra (KB-UZ/20–9/2021; 27 September 2021). Informed consent was obtained from all subjects involved in the study.

## 3 Results

### 3.1 Participant demographics

Three focus group discussions (FGDs) were carried out with adult UMs in December 2021 in Zielona Gora (with 7, 3, and 6 participants respectively) and another three FGDs — in January 2022 in Lublin (with 6 participants each); two other meetings with adolescent Ukrainian girls were conducted in January 2022 in Lublin (with 6 participants in each group). The mean age of the participants was 31.2 years. The mean duration of stay was 3.2 years: 40.0% of participants had resided in Poland for 2 years or less, 40.0% from 3 to 5 years, and 20.0% more than 5 years. Teenage girls, all high school students, represented 26.7% of all participants; adult UFMs who were high school graduates represented 26.7% of participants, while 44.4% were bachelor’s or master’s degree holders; 51.1% participants originated from Ukrainian cities >150,000 inhabitants, 35.6% from smaller cities, and the rest from rural areas. All adult UFMs had children. Two thirds of participants (66.7%) lived in the Polish city of Lublin and the rest in Zielona Gora.

### 3.2 Focus groups’ discussions

Three main themes, adapted from the Vaccine Hesitancy Determinants Matrix (VHM) constructs (24), were set up: (1) contextual influences, (2) individual and group influences, (3) vaccine/vaccination specific issues (Supplementary Table S2). Results of the FGD discussions presented in this subsection were organized by describing main themes, sub-themes/categories and the most important quotations (Supplementary Table S2). The study findings were put together to systematize complex, inter-dependent factors influencing COVID-19 vaccine decision-making process among economic UFMs in Poland. Factors were divided into two groups in terms to present this process from the Polish and Ukrainian perspective. Regarding some sub-themes, influencing factors overlapped and were presented as common for two countries.

#### 3.2.1 Contextual influences

We identified subthemes and categories within the main theme as contextual factors influencing UFMs’ decision to get the COVID-19 vaccine as follows:

- Culture/religion

Ukrainian female migrants reported that their own culture influenced their decision-making process. Participants confirmed that mothers were the primary decision maker regarding vaccinations in the Ukrainian family. One mother explained “*when it comes to the decisions, it’s all on me*” (female, age 35, FG7). Participating females were also asked about fathers’ role in the COVID-19 vaccination decision-making process. According to participants, a father, rather than taking on a part of the responsibility from a mother, was eagerly relating on her and was not much involved with the process.

Notably, Ukrainian orthodox religious groups were commonly mentioned has having a negative impact on the migrants’ COVID-19 vaccination decisions. “*People do not want to get vaccinated due to their religious beliefs…*” (female, age 42, FG 6).

- Communication and media environment

Ukrainian female migrants mentioned that mandatory vaccination-in place for routine childhood vaccines (but not COVID-19) made decision-making easier. “*Previously, there used to be a vaccination schedule with compulsory vaccines and we got our kids vaccinated according to the doctor’s advice. That was obligatory and everyone followed, now we have too much information…*” (female, age 33, FG 6). Some UFMs complained about the lack of consistency and confusion arising from multiple sources of information, from the internet or friends. Exposed to contradictory messages, some UFMs reported focusing on more authoritative resources, such as research papers. Some participants claimed to be “*reading into different experiences*” or “*resorting solemnly to medical resources*” (females, age 43, FG 3). Participants recognized the importance of objective, easily accessible data to make decisions about COVID-19 vaccines, and valued factual information, including statistical data, and trusted sources to make informed decisions.

- Policies – vaccine administration

Participants contrasted their experience of COVID-19 vaccination in Poland and Ukraine. Specifically, participants complained that in Ukraine “*there is no one who would run a proper health check or some tests*” (female, age 32, FG 1) before vaccination. UFMs saw such a health check as important, in particular in patients with comorbidities. Participants also voiced concerns about the lack of monitoring and reporting of adverse event following immunization in Ukraine. UFMs “*prefer to come to Poland to get vaccinated for COVID*” (female, age 47, FG 7), and negative experiences acquired in Ukraine in the past, may influence their decision process with regards to getting vaccinated against COVID-19 in Poland.

- Employment

Some UFMs complained that Polish employers sometimes directly or indirectly forced them to get vaccinated against COVID-19. As one participant explained “*they do not even provide one with choice*” (female, age 32, FG 6). Participants were also concerned that their autonomy around informed decision-making was not respected.

- Accessibility of the vaccine – language/product

Even for UFMs fluent in Polish, the technical language used when giving COVID-19 vaccines was a barrier. As one 42 years old female participant stated, they “*cannot recognize some of the medical terms*” (FG 1). Some UFMs asked relatives or friends fluent in Polish to accompany them when getting vaccinated. UFMs expressed preference for the “*proper*” vaccines (female, age 26, FG 1) made in the United States or in the Western Europe, as opposed to Chinese or Indian vaccines generally provided in Ukraine. Participants expressed doubt about the effectiveness of the latter ones. “*The only European one* (available COVID-19 vaccine) *is Pfizer. Otherwise, some Chinese and Indian ones are available. Even CureVac was ineffective*” (female, age 42, FG 1).

- Concern for differential treatment

UFMs complained about receiving discriminatory treatment, receiving an inferior service compared to their Polish. UFMs claimed that “*equal treatment is more important*” (female, age 42, FG 1) than other factors influencing decision-making.

#### 3.2.2 Individual and group influences

In this section relevant subthemes and quotations within the main theme, i.e., influences on the individual and group level were organized as follows:

- Personal/altruistic motivations

Fear of infection was reported as a reason for vaccination among some UFMs. The belief in the vaccine’s ability to protect against SARS-CoV-2 was an enabler to vaccination as was the desire to protect themselves from the severe consequences of COVID-19 and to make “*the illness be lighter*” (female, age 40, FG 8). Some UFMs expressed altruistic vaccination motivations, including how vaccinating themselves contributed to the fight against SARS-CoV-2. UFMs mentioned the desire to “*suppress the amount of infection*” and “*return to pre-pandemic state*” (female, age 42, FG 5). It was also emphasized that besides combating the pandemic, ending to the broader accompanying social impact was equally important, and vaccination was seen as an important tool for this.

- Social influences

Multiple interviewees mentioned that decisions on COVID-19 vaccination were influenced by friends and relatives within their close social circles. UFMs reported that improved attitudes and increased intention to get vaccinated, was based on positive experiences of other family members. One UFMs stated that seeing some close friends getting vaccinated against COVID-19 increased her confidence in the vaccine and made her decide to vaccinate. Conversely, negative experiences of others worsened UFMs’ attitudes and motivation toward vaccination.

- Health system and providers – trust and personal experience

The UFMs reported higher levels of trust in the Polish healthcare system and providers compared to the Ukrainian system. Many participants mentioned preferring to come to Poland to get vaccinated for COVID-19, based on their personal experience with the Ukrainian system: “*I know that whatever they have in Ukraine will not work*” (female, age 37, FG 2).

- Return to routine

Beyond health concerns, most UFMs viewed vaccination pragmatically as a way to return to routine and to “*make their lives simpler*” (female, age 43, FG 6). The travel related issues, such as dealing with documentation at the border while traveling to school, work etc., were also very important factors in COVID-19 vaccination decision-making. A 40 year old female commented as follows: “*I got vaccinated for practical reasons, to travel in and out of the country*” (FG 8).

- Influence of other vaccines

UFMs expressed how negative experiences with COVID-19 vaccines reported in Ukraine impacted their decision regarding child vaccination, and *vice-versa*. A 35-year old Ukrainian female stated that in Ukraine, there were cases where “*after getting certain vaccines, children experienced serious side effects*” (FG 2), and she was concerned the same could happen after COVID-19 vaccination. Parents and grandparents often delayed or postponed COVID-19 vaccination in their children or grandchildren, citing a lack of concrete scientific evidence as their main concern.

#### 3.2.3 Vaccine or vaccination – specific issues (directly related to vaccine or vaccination)

In the last section subthemes and quotations related to vaccine or vaccination were highlighted.

- Unnecessary/ineffective vaccine

The UFMs reported they did not see a compelling reason for getting vaccinated. A 45 year-old female migrant (FG 1) asked “*Why would we?*.” Another one claimed that COVID-19 vaccine “*does not do anything good*” (female, age 43, FG 3). Several participants mentioned pressure or compulsion to vaccinate as the main reason for going through with the vaccination procedure.

- Concerns about vaccine development

The UFMs questioned the rapid vaccine development process. A 37 year-old female (FG 1) asked, “*How come they invented it within just a year?*.” The short time frame led to migrants questioning whether the available vaccines had been sufficiently researched.

- Vaccine safety and efficacy

A lot of Ukrainians did not want to be vaccinated because they doubted COVID vaccines were effective or even genuine. “*They think it’s just some kind of water or something…*” (female student, age 16, FG 5). The perceived lack of efficacy led to participants querying their necessity. Some UFMs went as far as wondering whether the vaccines were harmful as a result of some of the ingredients they thought the vaccines contained, such as metals or formalin. Disinformation about COVID-19 vaccine side effects, including risk of death, negatively impacted migrants’ decision-making process. According to participants, Ukrainian media relayed myths and misconceptions about COVID-19 vaccines. Conspiracy opinions, such as the existence of operations to cover-up vaccine related deaths, also existed: “*if they were vaccinated and then die, it’s never because of the vaccine obviously*” (female, 40, FG 1).

- Vaccine storage

Participants expressed doubts adequate storage and handling in Ukraine. Pictures circulating on social media among Ukrainian groups showed that “*COVID vaccines were kept in direct sunlight.*” (female, age 26, FG 8). Most Ukrainians did not want to get vaccinated with such “*questionable products*” (female, age 40, FG 7).

- Vaccine cost

The UFMs noted that while the availability of COVID-19 vaccines produced in the Western Europe or the US was scarce, it was still possible to purchase them outside the system. Traveling abroad to get vaccinated was mentioned as a common practice, with a particular preference for EU countries, where vaccines were believed to be safer and of better quality. A 43 year-old female explained: “*We got vaccinated for COVID in Poland, yet with European vaccines, different to the ones widely available in Ukraine. I paid for them.*” (FG 1). Respondents also mentioned purchasing fake COVID-19 certificates (to avoid vaccination) as a common practice among their fellow Ukrainians. Prices for forged certificates seemed to widely fluctuate. While some of the interviewees claimed this practice was not as prevalent as it used to be, most said that it was still largely popular, especially among those who wanted to travel without constraints without getting vaccinated. A 35-year old female claimed “*a lot of people, my own friends, purchased those certificates just to be able to travel abroad*” (FG 2).

- Trust in practitioners’ recommendations

Interviewees claimed that they placed a lot of trust in GPs’ recommendations on COVID-19 vaccination. UFMs overwhelmingly described Polish GPs as a trusted source of information and stated that they “*like research based advice, not one from a person who will make something up.*” (female, age 42, FG 8). UFMs claimed GPs were especially influential in their decision-making process. While most participants reported the positive influence of Polish health professionals, others described lack of professionalism universally present in Ukrainian healthcare (“*you can run into a doctor that knows less than you do*”; female, age 37, FG 7), as well as bribery (“*all those [doctors] over 40 were schooled to partake in the bribe scheme*”; female, age 42, FG 7).

## 4 Discussion

To the authors knowledge this is the third qualitative study focusing on vaccination among Ukrainian migrants residing in Poland. Previously conducted studies examined the general attitudes toward the vaccines (8) and the structural barriers in access to the vaccines (21). This study looks specifically at UM’s barriers and enablers toward COVID-19 vaccination and factors underlying the vaccination decision-making.

The study, based on FGDs with UFMs in Poland, was conducted just before the Russian aggression toward Ukraine. Therefore, it mainly reflects the views of Ukrainian economic migrants. Key analysis themes which emerged from the qualitative data, with the help of the Working Group Determinants of Vaccine Hesitancy Matrix, were related to contextual, individual/group and the COVID-19 vaccine and vaccination influences. In terms of contextual influences arising from cultural factors, matrilineal culture was found to be playing an important role in the vaccine decision-making process among UFMs. In this culture, older women are teachers and holders of traditional knowledge and they are entitled to and entrusted with creative roles, including adaptation to changing conditions, such as vaccination decisions and migration (25). Our study confirmed the influence of matrilineal decision makers regarding COVID-19 vaccination on child and adolescent immunization. Cultural norms influenced community members’ decisions to get the COVID-19 vaccine after mothers had requested it. Thus the importance of getting females and mothers on board should be taken into consideration when planning vaccination strategies in context where such a matrilinear culture is important, as has been observed in other contexts (26). The influence of female on their partners (as opposed to their children) with regards to vaccination decision needs further investigation.

The UFMs reported differences in the perceived quality of healthcare services in Ukraine and Poland, with these experiences shaping their confidence in health interventions including vaccines. Participants blamed the Ukrainian state structures for not providing sufficient care to the citizens, including vaccines. The confidence in the Ukrainian government was low, as the population witnessed various political crises in the pre-war period (27). UFMs reported higher levels of trust in the Polish healthcare system, to the extent that in some instances Ukrainians came to Poland to get vaccinated against COVID-19. Some studies have highlighted the importance of rebuilding trust in state institutions in order to positively influence vaccination decision-making (28, 29).

In our study, as well as in the literature, compulsory vaccination or vaccination as a condition to employment was a strong enabler among participants wanting to preserve jobs (30, 31), but the perception was mixed. Notably, participants complained that through pre-established, strict rules employers were forcing them to get the COVID-19 vaccine despite their hesitancy. Additionally, some studies report the dilemma between potentially losing jobs and getting vaccinated, in cases where vaccination was not done by personal choice (31). This observation suggests that unemployed Ukrainian migrant could have less incentives to vaccinate, creating inequalities within the migrant population.

Consistent with prior COVID-19 vaccine acceptability research (26, 32, 33), we found the desire to return to normal activities as another influential factor in UFMs’ decision-making. For many migrants, getting back to normal was synonym with being able to travel abroad. This finding was consistent with finding from another study that showed that avoidance of “travel ban” was one of the major predictors behind COVID-19 vaccination (32).

Personal factors, such as fear of infection or COVID-19 illness, and altruistic motivations, for instance contributing to eliminating COVID-19 or combating the virus together were also found as the motivators for getting vaccinated. Some UFMs described knowledge and awareness of negative COVID-19 outcomes among their family members and social network, as well as coworkers, as potential barriers to COVID-19 vaccination decision-making. A similar phenomenon was also observed in other studies (24, 33, 34). Unlike other studies (33, 35) family and social pressure to vaccinated was not mentioned. Our findings support that influences arising from the personal and immediate and wider social circles can influence the decision to get vaccinated. Additionally, we provide the important message that UFMs’ share decision-making with family, friends and coworkers.

Ukrainian female migrants expressed a high level of trust in the Polish healthcare system and healthcare providers; such trust if properly leveraged can improve willingness to vaccinate against COVID-19 (35–37). Indeed professional recommendations from healthcare providers can improve intention to vaccinate (8, 26, 28, 34–36, 38, 39). In our study, Polish healthcare providers, as well as those with Ukrainian background working in Poland, can play a crucial role in improving trust. Our study shows that while trust in the Ukrainian health system is low, trust in the healthcare system among migrants increases after migration from Ukraine to Poland. Such trust should be leveraged, especially as migrant populations are less likely to receive physician recommendations for vaccinations (28). Evidence also suggests that having had a negative previous experience with vaccines also decreases future intention to vaccinate (40). Thus potential pre-migration negative experiences around vaccines in Ukraine may influence vaccine attitudes and decision-making after arriving to Poland.

Consistent with published evidence (27, 36, 40, 41), we found concerns about the vaccine’s rapid development and a lack of scientific evidence as barriers to immunization. Specifically, UFMs did not trust the short vaccine development process, expressing serious concerns that these vaccines might have unknown short and long-term adverse effects. Our results support previous reports (40, 42) that these concerns influence the decision to get vaccinated. Participants however distinguished between COVID-19 vaccines in Ukraine, seen to be ineffective and potentially dangerous, and vaccines provided in Poland, perceived as being of higher quality. This was similar to a previous paper on immunization among Ukrainian migrants (8), showing that compared to Ukraine, vaccines provided in Poland were seen as being manufactured by trusted, well recognized European or American brands, and were appropriately stored and administered. UFMs believed that side effects observed in Poland were much less numerous than compared to Ukraine. To our knowledge, this is the first qualitative study to document the perceived difference in COVID-19 vaccine quality, safety and effectiveness between Poland and Ukraine, described in participants’ own words.

### 4.1 Strengths and limitations

One strength of our study was a diverse sample that included UFMs of different demographic characteristics, residing in several different Polish regions. Despite the relatively low sample size, a limitation of qualitative studies (43, 44), the authors believe that data saturation was reached in this sample of migrants. However, Ukrainian migrants to Poland are diverse in terms of backgrounds and socio-demographic characteristics and the study may not representative of UFMs from other parts of the country. The inclusion of two geographically different regions of Poland might reduce this bias. Women are the key decision-maker for the family with regards to vaccine and for this reason we focused on this group. Findings may be different among men and holding focus groups with male UFMs may be of value. While the snowball sampling used in this study may limit generalizability to the larger UFMs population (8), our sample included a range of ages, literacy, areas of origin in Ukraine, and length of stay in Poland. As such, relatively heterogeneous opinions could be obtained. Finally, the vaccine hesitancy matrix may be not free from limitations when applied to a novel vaccine such as COVID-19. Earlier evaluation suggest the matrix is fit for evaluation of COVID-19 vaccines. Of note, COVID-19 vaccine awareness and knowledge has changed over the course of the pandemic (28). Since our interviews took place prior to the 2022 Russian attack on Ukraine, we cannot measure the impact of the War on vaccine perception; likewise we cannot generalize our finding to the war refugees, a more highly educated population than economic migrants.

## 5 Conclusion

Barriers to vaccination among UFMs were related to contextual influences, individual and group level influences and vaccine- or vaccination-specific issues. The results are consistent to existing evidence reported in the scientific literature (8, 26, 28, 31, 33, 39, 45). While some of the factors we identified had been previously identified, this study also brings new insights. First, most participants did not consider their personal and communal interests as major factors in decision-making. Second, the novelty of the vaccine and a consequent fear of poorly studied adverse effects and poor effectiveness were major factors. Third, the perceived need for immunization was not universal. Fourth, negative experiences with vaccines provided in Ukraine, specifically childhood vaccines, highlight possible links between experience of past vaccination and perceptions of risk from the COVID-19 vaccine, and elements of hesitancy formed in the country of origin may persist after migration: this might be another barrier to vaccination among migrants. This brings a new perspective not only applicable to COVID-19 immunization in Poland, but also generalizable to other countries hosting UFMs, as well as to other non-mandatory vaccines. By identifying factors enabling COVID-19 vaccine decision-making among the Ukrainian migrant community, the present study provides evidence that may inform the development of adequate tailored strategies to limit vaccine hesitancy.

Such strategies can include, beyond broad national COVID-19 vaccine campaigns, tailored communication campaigns and approaches, focused on the Ukrainian community in Poland; training Ukrainian healthcare providers working in Poland and Ukrainian community leaders, specifically females, could be crucial. Trained individuals could then act as vaccine ‘role models’ who can discuss vaccination and address misinformation. In addition, health care provider-Ukrainian patient interpersonal communication play a pivotal role especially when trust in Polish healthcare workers is high. Motivational, vaccine-oriented conversation could help Ukrainian migrants make the informed decision to vaccinate.

## Data Availability

The raw data supporting the conclusions of this article will be made available by the authors, without undue reservation.

## References

[1] World Health Organization COVID-19 Dashboard. Number of COVID-19 cases and deaths (cumulative total). Available at: https://data.who.int/dashboards/covid19/cases?n=c (accessed January 10, 2024)

[2] Carabelli AM Peacock TP Thorne LG Harvey WT Hughes J COVID-19 Genomics UK Consortium. SARS-CoV-2 variant biology: immune escape, transmission and fitness. Nat Rev Microbiol. (2023) 21:162–77. doi: 10.1038/s41579-022-00841-7, PMID: 36653446 PMC9847462

[3] Saag M. Wonder of wonders, miracle of miracles: the unprecedented speed of COVID-19 science. Physiol Rev. (2022) 102:1569–77. doi: 10.1152/physrev.00010.2022, PMID: 35446679 PMC9169823

[4] World Health Organization. COVID-19 advice for the public: getting vaccinated. Available at: https://www.who.int/emergencies/diseases/novel-coronavirus-2019/covid-19-vaccines/advice (accessed December 5, 2023)

[5] Trackvaccines.org. Available at: https://covid19.trackvaccines.org/country/ukraine/ (accessed October 14, 2022)

[6] Rzymski P Falfushynska H Fal A. Vaccination of Ukrainian refugees: need for urgent action. Clin Infect Dis. (2022) 75:1103–8. doi: 10.1093/cid/ciac276, PMID: 35435230 PMC9383728

[7] Holt E. COVID-19 vaccination in Ukraine. Lancet Infect Dis. (2021) 21:462. doi: 10.1016/S1473-3099(21)00156-0, PMID: 33773128 PMC7990473

[8] Ganczak M Bielecki K Drozd-Dąbrowska M Topczewska K Biesiada D Molas-Biesiada A. Vaccination concerns, beliefs and practices among Ukrainian migrants in Poland: a qualitative study. BMC Public Health. (2021) 21:93. doi: 10.1186/s12889-020-10105-9, PMID: 33413287 PMC7789884

[9] Vojtek I Larson H Plotkin S Van Damme P. Evolving measles status and immunization policy development in six European countries. Hum Vaccin Immunother. (2022) 18:2031776. doi: 10.1080/21645515.2022.2031776, PMID: 35180372 PMC9009904

[10] Matiashova L Isayeva G Shanker A Tsagkaris C Aborode AT Essar MY. COVID-19 vaccination in Ukraine: an update on the status of vaccination and the challenges at hand. J Med Virol. (2021) 93:5252–3. doi: 10.1002/jmv.27091, PMID: 34002400 PMC8242568

[11] Patel SS Moncayo OE Conroy KM Jordan D Erickson TB. The landscape of disinformation on health crisis communication during the COVID-19 pandemic in Ukraine: hybrid warfare tactics, fake media news and review of evidence. J Sci Commun. (2020) 19:A02. doi: 10.22323/2.19050202, PMID: 34504624 PMC8425291

[12] Broniatowski DA Jamison AM Qi S AlKulaib L Chen T Benton A. Weaponized health communication: twitter bots and Russian trolls amplify the vaccine debate. Am J Public Health. (2018) 108:1378–84. doi: 10.2105/AJPH.2018.304567, PMID: 30138075 PMC6137759

[13] Odarchenko K.. Wilson Center Vaccine hesitancy in Ukraine: the sign of a crisis in governance? | Wilson Center. Available at: https://www.wilsoncenter.org/blog-post/vaccine-hesitancy-ukraine-sign-crisis-governance (accessed May 14, 2022)

[14] Bazylevych M. Vaccination campaigns in postsocialist Ukraine: health care providers navigating uncertainty: vaccination campaigns in postsocialist Ukraine. Med Anthropol Q. (2021) 25:436–56. doi: 10.1111/j.1548-1387.2011.01179.x, PMID: 22338289

[15] Cambridge English Dictionary, p. economic migrant. Available at: http://cambridge.english.dictionary:meanings&definitions (accessed June 25, 2024)

[16] Górny A., New dimensions in immigration from Ukraine to Poland. CMR Spotlight (2019) 9. (Accessed 25 June, 2024).

[17] Chmielewska-Kalińska I Dudek B Strzelecki P. The living and economic situation of Ukrainian refugees in Poland Report of the questionnaire survey conducted by NBP Regional Branches. (2022). Available at: https://nbp.pl/wp-content/uploads/2022/11/ukrainian-refugees-2022.pdf (accessed October 27, 2023)

[18] Malchrzak W Mastalerz-Migas A Sroka Z Spiegel M. One year of the COVID-19 pandemic. What do we know and what is yet to come? — the summarising review. Int J Public Health. (2021) 66:66. doi: 10.3389/ijph.2021.1603975, PMID: 34588946 PMC8475762

[19] Troiano G Torchia G Nardi A. Vaccine hesitancy among Ukrainian refugees. J Prev Med Hyg. (2022) 63:E566–72. doi: 10.15167/2421-4248/JPMH2022.63.4.2774 36890995 PMC9986985

[20] RIVER-EU. Available at: https://eupha.org/RIVER-EU (accessed October 27, 2023)

[21] Ganczak M Kalinowski P Pasek O Duda-Duma Ł Sobieraj E Goławski J. Health system barriers to child mandatory and optional vaccination among Ukrainian migrants in Poland in the context of MMR and HPV vaccines-a qualitative study. Int J Environ Res Public Health. (2022) 20:712. doi: 10.3390/ijerph20010712, PMID: 36613034 PMC9819946

[22] Daniel BS Murrell DF. The role of women as past and present advocates for vaccinations: relevance in the COVID-19 setting. Int J Women's Dermatol. (2021) 7:228–9. doi: 10.1016/j.ijwd.2020.10.001, PMID: 33195788 PMC7648508

[23] Braun V Clarke V. Using thematic analysis in psychology. Qual Res Psychol. (2006) 3:77–101. doi: 10.1191/1478088706qp063oa

[24] MacDonald NE. Vaccine hesitancy: definition, scope and determinants. Vaccine. (2015) 33:4161–4. doi: 10.1016/j.vaccine.2015.04.036, PMID: 25896383

[25] Guédon MF. An introduction to Matri-culture, the concept. Matrix J Matricult Stud. (2020) 1:3–7.

[26] Purvis RS Moore R Willis DE Hallgren E McElfish PA. Factors influencing COVID-19 vaccine decision-making among hesitant adopters in the United States. Hum Vaccin Immunother. (2022) 18:2114701. doi: 10.1080/21645515.2022.2114701, PMID: 36070518 PMC9746519

[27] Kukuła A. Political, social and economic conditions of development of contemporary Ukraine and its regions. Lublin: Wydawnictwo KUL (2016).

[28] Carson SL Casillas A Castellon-Lopez Y Mansfield LN Morris D Barron J. COVID-19 vaccine decision-making factors in racial and ethnic minority communities in Los Angeles, California. JAMA Netw Open. (2021) 4:e2127582. doi: 10.1001/jamanetworkopen.2021.27582, PMID: 34591103 PMC8485164

[29] Daniels D Imdad A Buscemi-Kimmins T Vitale D Rani U Darabaner E. Vaccine hesitancy in the refugee, immigrant, and migrant population in the United States: a systematic review and meta-analysis. Hum Vaccin Immunother. (2022) 18:2131168. doi: 10.1080/21645515.2022.2131168, PMID: 36332155 PMC9746503

[30] Ahmad Malik J Ahmed S Shinde M Almermesh MHS Alghamdi S Hussain A. The impact of COVID-19 on comorbidities: a review of recent updates for combating it. Saudi J Biol Sci. (2022) 29:3586–99. doi: 10.1016/j.sjbs.2022.02.006, PMID: 35165505 PMC8828435

[31] Blahut R Flint A Orlando E DesChatelets J Khowaja A. A scoping review on the decision-making dynamics for accepting or refusing the COVID-19 vaccination among adolescent and youth populations. BMC Public Health. (2023) 23:784. doi: 10.1186/s12889-023-15717-5, PMID: 37118794 PMC10141871

[32] Morales GI Lee S Bradford A De Camp A Tandoc EC Jr. Exploring vaccine hesitancy determinants during the COVID-19 pandemic: an in-depth interview study. SSM Qual Res Health. (2022) 2:100045. doi: 10.1016/j.ssmqr.2022.100045, PMID: 35128519 PMC8800497

[33] Fojnica A Osmanovic A Đuzic N Fejzic A Mekic E Gromilic Z. COVID-19 vaccine acceptance and rejection in an adult population in Bosnia and Herzegovina. PLoS One. (2022) 17:e0264754. doi: 10.1371/journal.pone.0264754, PMID: 35226708 PMC8884480

[34] Gogoi M Wobi F Qureshi I Al-Oraibi A Hassan O Chaloner J. “The vaccination is positive; I don't think it's the panacea”: a qualitative study on COVID-19 vaccine attitudes among ethnically diverse healthcare workers in the United Kingdom. PLoS One. (2022) 17:e0273687. doi: 10.1371/journal.pone.0273687, PMID: 36084076 PMC9462779

[35] Ledford CJW Cafferty LA Moore JX Roberts C Whisenant EB Garcia Rychtarikova A. The dynamics of trust and communication in COVID-19 vaccine decision making: a qualitative inquiry. J Health Commun. (2022) 27:17–26. doi: 10.1080/10810730.2022.2028943, PMID: 35220915

[36] Abba-Aji M Stuckler D Galea S McKee M. Ethnic/racial minorities’ and migrants’ access to COVID-19 vaccines: a systematic review of barriers and facilitators. J Migr Health. (2022) 5:100086. doi: 10.1016/j.jmh.2022.100086, PMID: 35194589 PMC8855618

[37] Badur S Ota M Öztürk S Adegbola R Dutta A. Vaccine confidence: the keys to restoring trust. Hum Vaccin Immunother. (2020) 16:1007–17. doi: 10.1080/21645515.2020.1740559, PMID: 32298198 PMC7227637

[38] Hussain B Latif A Timmons S Nkhoma K NellUFMs LB. Overcoming COVID-19 vaccine hesitancy among ethnic minorities: a systematic review of UK studies. Vaccine. (2022) 40:3413–32. doi: 10.1016/j.vaccine.2022.04.030, PMID: 35534309 PMC9046074

[39] Naqvi M Li L Woodrow M Yadav P Kostkova P. Understanding COVID-19 vaccine hesitancy in ethnic minorities groups in the UK. Front Public Health. (2022) 10:917242. doi: 10.3389/fpubh.2022.917242, PMID: 35844884 PMC9284000

[40] European Centre for Disease Prevention and Control. Let’s talk about hesitancy. Stockholm: ECDC (2016).

[41] Freeman D Loe BS Chadwick A Vaccari C Waite F Rosebrock L. COVID-19 vaccine hesitancy in the UK: the Oxford coronavirus explanations, attitudes, and narratives survey (oceans) II. Psychol Med. (2022) 52:3127–41. doi: 10.1017/s0033291720005188, PMID: 33305716 PMC7804077

[42] Robertson E Reeve KS Niedzwiedz CL Moore J Blake M Green M. Predictors of COVID-19 vaccine hesitancy in the UK household longitudinal study. Brain Behav Immun. (2021) 94:41–50. doi: 10.1016/j.bbi.2021.03.008, PMID: 33713824 PMC7946541

[43] Gorman DR Bielecki K Willocks LJ Pollock KG. A qualitative study of vaccination behaviour amongst female polish migrants in Edinburgh, Scotland. Vaccine. (2019) 37:2741–7. doi: 10.1016/j.vaccine.2019.03.073, PMID: 30979570

[44] Francis JJ Johnston M Robertson C Glidewell L Entwistle V Eccles MP. What is an adequate sample size? Operationalizing data saturation for theory-based interview studies. Psychol Health. (2010) 25:1229–45. doi: 10.1080/08870440903194015, PMID: 20204937

[45] Deal A Crawshaw AF Carter J Knights F Iwami M Darwish M. Defining drivers of under-immunization and vaccine hesitancy in refugee and migrant populations. J Travel Med. (2023) 30:aad084. doi: 10.1093/jtm/taad084, PMID: 37335192 PMC10481413

