# Supplementary material for: “Why would we?” A qualitative study on COVID-19 vaccination decision making among Ukrainian economic female migrants in Poland
Source: Front Public Health. 2024 Aug 13;12:1380627. doi: 10.3389/fpubh.2024.1380627 (PMC11347284; doi:10.3389/fpubh.2024.1380627)
Supplement: Supplementary file 1 [file Table_1.DOCX]

Supplementary Material

## Supplementary Tables

| **Theme** | **Sub-theme** |
| --- | --- |
| Contextual influences  Influences arising due to historic, socio-cultural, environmental, health system/institutional factors | Culture/Religion |
|  | Policy |
|  | Communication and media environment |
| Individual and group influences  Influences arising from personal perception of the vaccine or influences of the social/peer environment | Beliefs and attitudes about health, prevention and vaccination; conspiracy theories |
|  | Personal, family and community members’ experience with vaccination |
|  | Health system and providers – trust and personal experience |
|  | Immunization not needed |
| Vaccine or Vaccination specific issues  Directly related to vaccine or vaccination | Development of a new vaccine |
|  | Reliability / source of supply |
|  | Costs |
|  | The strength of the recommendation / attitudes of healthcare professionals |

**Supplementary Table 1.** Selected key analysis themes and sub-themes emerged from the qualitative data, according to the *Working Group Determinants of Vaccine Hesitancy Matrix* [21].

| **Theme / sub-theme** | **Quotation** | |
| --- | --- | --- |
| **Contextual influences arising from sociocultural, historical, health system or institutional factors** | | |
|  | In Poland | In Ukraine |
| Matrilineal culture | *“It was really me who was the decision-maker* [regarding vaccinations]. *My husband believed in all my choices. I’m a nurse so he was saying that ‘If you say that we should do it, that’s what we are going to do’* (female, age 36)  *Then it was also me, as my husband claims that I know our children better and he doesn't want to interfere. He trusts me.* [in in relation to vaccination]. (female, age 43) | |
| Religious groups endorsing vaccination prohibition |  | *“**And here the opinion on COVID can differ. From one side, vaccines are a health-related necessity, and from the other side, it’s not a necessity due to the religious beliefs*.” (female age 40)  *“I haven’t heard much about instances as such, yet, there are Ukrainian religious organizations, which prohibit vaccines.”* (female, age 43) |
| Imprecise/Untrustworthy/ Confusing Information | *“Previously, there used to be a vaccination schedule with compulsory vaccines and me and my parents got our kids vaccinated according to the doctor's advice. That was obligatory and everyone followed, now we have too much information from the internet or friends.”*(female, age 43)  “*When it comes to vaccines, I don’t read anything on Facebook. I’m reading into different experiences. I have access to various media outlets. I’m reading research papers from the US, Canada or Israel. I resort solemnly to medical resources.* (female, age 42)  *“I could be persuaded* [to get vaccinated for COVID-19] *with a movie that would include some statistical data, with sources given.”*(female, age 29) |  |
| Accessibility barriers: language (Poland) / vaccine (Ukraine) | *“You visit a doctor who uses medical terminology and technically you were there but you still don’t know anything and end up resorting to online resources.”*(female, age 35)  *“I know Polish quite well, yet sometimes, I cannot recognize some of the medical terms. Thus, for example, when I visit a doctor to have my child, or myself, vaccinated*, *I would want to receive a leaflet about possible side effects and what to expect.”* (female, age 42)  *“If they* [vaccine providers] *could redirect you to a doctor, with whom you can actually speak Ukrainian...”*(female, age 40) | *“In Ukraine, it’s really hard to access proper* [COVID-19] *vaccines.”*(female, age 26)  [From the available COVID-19 vaccines], *“the only European one is Pfizer. Otherwise, some Chinese and Indian ones are available. Even CureVac was ineffective.”*(female, age 42) |
| Communication and media environment | *“Previously, there used to be a vaccination schedule with compulsory vaccines and me and my parents got our kids vaccinated according to the doctor's advice. That was obligatory and everyone followed, now we have too much information from the internet or friends.”* (female, age 33) | |
| Trust in the vaccine administration | *“Many [Ukrainians] prefer to come to Poland to get vaccinated for COVID here.”* (female, age 47) | *“I just don't believe that the vaccines will be properly administered in Ukraine.”* (female, age 25)  *“I believe that vaccines available in Ukraine are fine, yet the government doesn’t provide sufficient care to their citizens. If someone has various health issues and gets vaccinated, of course it can be fatal. But there is no one who would run a proper health check or some tests* [on him]. *They just go with the procedure. It’s not about the vaccines but rather about the country.”*(female, age 32)  *“I think we* [Ukrainians] *simply don’t believe in state structures. And since we don’t, people also end up not trusting doctors.”*(female, age 40) |
| Employment | *“Drivers are technically allowed to test* [for SARS-CoV-2 infection] *but it’s not possible for us to be driving even during the evenings and on top of that, get tested daily. Thus, all drivers, even though we are in the EU, virtually have no choice* [for SARS-CoV-2 test] *and get vaccinated.”*(female, age 36)  *“In some places, they don’t even provide one with choice. They will not directly force you to do it [COVID-19 vaccination] but essentially leave you no choice.”* (female, age 32)  *“Unless they [employers] order so, then this* [COVID-19 vaccination] *is obligatory.”*(female student, age 17) | |
| **Individual and group influences (arising from the personal/social/peer environment perception of the COVID-19 vaccine)** | | |
| Personal motivations | *“I got vaccinated as I think that if I get sick one day, the illness will be lighter.”* (female*,* age 40*)*  *“I got vaccinated since I went through COVID twice.”* (female, age 32) | |
| Altruistic motivations | *[I got vaccinated against COVID] “to suppress the amount of infections. Also, I hope the world will soon return to a pre-pandemic state, or it will be just like in the beginning of the pandemic. So we can all travel without constraints and I hope we can access other countries again.”*(female, age 42)  *“I got vaccinated against COVID since if all adults do so, we can combat the virus together. I’m still debating on vaccinating my child, however.”*(female, age 25) | |
| Social learning patterns | *“There are various reasons for which I was vaccinated, first - my entire family is vaccinated…”* (female, age 45)  *“I’m using the experience of my closest family members. When I got vaccinated with Pfizer, I felt fine, just like my husband did.”*(female, age 45)  *“Also, some friends… If a friend gets vaccinated* [for COVID-19] *and tells me that it’s a proper thing, then I can get vaccinated as well.”*(female, age 35) | |
| Free to return to normal | *“The great majority has a rather practical approach when it comes to COVID vaccine, that it will make our lives simpler and there won’t be as many infections, so it’s all going to be easier.”*(female, age 43)  *“At first I was against getting vaccinated myself, yet, later on, practical matters emerged when we all had to travel together due to school.”* (female student, age 16)  *“It’s more practical. Then dealing with* [COVID-19] *documentation at the border is easier.”*(female, age 37)  *“I got vaccinated for practical reasons, to travel in and out of the country.”*(female, age 40) | |
| Skepticism about child vaccination | *At some point, I was debating on getting the vaccine myself. When it comes to kids, I wasn’t really thinking of having them vaccinated. I’ve heard opinions that not everyone feels good after getting this vaccine.*female, age 35)  *All* [mandatory] *vaccines have been taken. Although, when it comes to the COVID vaccine, I’m a bit hesitant. I don’t know how it will influence a child like mine.*(female, age 33)  *I will get my eldest son vaccinated against COVID in a month or so. I’m a bit skeptical about having the younger one vaccinated.*(female, age 38) | *In Ukraine, there were cases where, after getting certain vaccines, children experienced serious side effects, and I’m afraid of this when it comes to the COVID vaccine.* (female, age 35) |
| Health system and providers – trust & personal experience | *Many [Ukrainians] prefer to come to Poland to get vaccinated for COVID-19 here.* (female, age 47)  *We got vaccinated for COVID-19 in Poland, yet with European vaccines, different to the widely available in Ukraine.* (female, age 43) | *From personal experience, I know that whatever they have in Ukraine won’t work.*(female, age 37) |
| Immunization not needed | *Nowadays, most people think that if you get vaccinated* [for COVID-19], *it won’t help you… that it doesn’t do anything good.* (female, age 43)  *They* [Ukrainians] *don’t get vaccinated thinking ‘Why would we?’. Our ancestors have lived long lives and weren’t dying and now, they get vaccinated and indeed they are dying.*(female, age 45) | |
| **Vaccine or vaccination – specific issues (directly related to vaccine or vaccination)** | | |
| Vaccine development misconception | *How come they invented the* [COVID-19] *vaccine within just a year?*(female, age 37)  *My point is that I don’t want to risk my child’s health and vaccinate her against COVID, since it’s not properly researched.* (female, age 29)  *Children are dying because of COVID vaccines. Maybe it means that it’s not properly researched? And now, they keep vaccinating people.*(female, age 31) | |
| Vaccine storage | *I have more trust in Polish vaccines and clinics, especially since I can see in what temperature everything is stored.*(female, age 45) | *People don’t trust the vaccines sold in Ukraine, they don’t trust they are stored as required.*(female, age 47)  *There were a lot of instances where the vaccines weren’t properly stored. On Facebook, there were a lot of pictures posted, showing vaccines kept in direct sunlight. . .*(female, age 26)  *And we don’t believe that they [vaccines] are stored properly. So Ukrainians don’t want to get vaccinated with questionable products.* (female, age 40) |
| Vaccine effectiveness/ quality/ safety | *… I’m not an anti-vaxer, my eldest got all required vaccines but I didn’t do the same with the younger one. I have my views, some of them against vaccinating for COVID. It can be that they use formalin. And I did not vaccinate him previously and my child is healthy.*(female, age 37)  *Well, we don’t know what ingredients, even metals, could be in this* [COVID] *vaccine.* (female, age 37) | *We don’t believe that vaccines are good, as in that the vaccines that the Ukrainian government purchased are good quality.* (female, age 40)  *I know for now, when it comes to vaccination, a lot of my colleagues from Ukraine do not want to be vaccinated. They're not sure it's really a COVID vaccination. They think it's just some kind of water or something...*(female student, age 16) |
| Vaccine side effects | *I’m postponing having my eldest vaccinated for COVID. He’s still in puberty, his voice is changing so I’m afraid of any possible negative effects of the vaccine on, e.g., him changing from a boy into a man.*(female, age 40)  *We don’t know much about its* [COVID vaccine] *effect in two or six years.*(female, age 37) | *In Ukraine, there were cases where, after getting certain vaccines, children experienced serious side effects, and I’m afraid of this when it comes to the COVID vaccine.* (female, age 35) |
| Vaccine cost  Vaccination cost |  | *I paid for them* [COVID vaccines]*. I don’t have a single vaccine that I didn't pay for.* (female, age 43)  *"Cheap’… that applied to COVID vaccines* (female, age 43)  *I know a few people that bought it* [COVID-19 certificate]. (female, age 43)  *Even among my friends, whom I know personally, they simply buy the certificates. Those who had the money for it bought it. It wasn’t even that expensive. (*female, age 40*)*  *There even was a man who came to Poland, in order to then, buy the certificates for his entire family. The money he earned here was taken to Ukraine to make this purchase.*(female, age 37) |
| Desire for practitioner recommendation | *When it comes to vaccines, I trust my GP the most. I like research based advice, not one from a person who will make something up.*(female, age 42)  *I ended up in my GP’s office. She advised me that the Pfizer vaccine would be the best choice. Besides, she mentioned that more people have less side effects since it's a second dose vaccine, now even third.* (female, age 42)  *For sure, GPs. They take care of healthcare related issues throughout our entire lives…* (female, age 29) | *I don't trust Ukrainian doctors since all those over 40 were schooled to partake in the bribe scheme. That’s why chances to stumble upon a normal doctor our age are slim.* (female, age 42)  *Sometimes you can run into a doctor that knows less than you do, even though you are not a doctor yourself. You can even see them googling what should be prescribed etc.. How could I trust them then?* (female, age 37)  *In Ukraine, I had less trust in doctors, even those that I was more or less sure off.* (female, age 36) |

**Supplementary Table 2.** Themes and sub-themes, important quotations for COVID-19 decision making within Ukrainian migrants in Poland using WHO Vaccine Hesitancy Matrix categories
